# Supplementary figures and images for: Atypical memory B-cells and autoantibodies correlate with anemia during Plasmodium vivax complicated infections
Source: PLoS Negl Trop Dis. 2020 Jul 20;14(7):e0008466. doi: 10.1371/journal.pntd.0008466 (PMC7392348; doi:10.1371/journal.pntd.0008466)

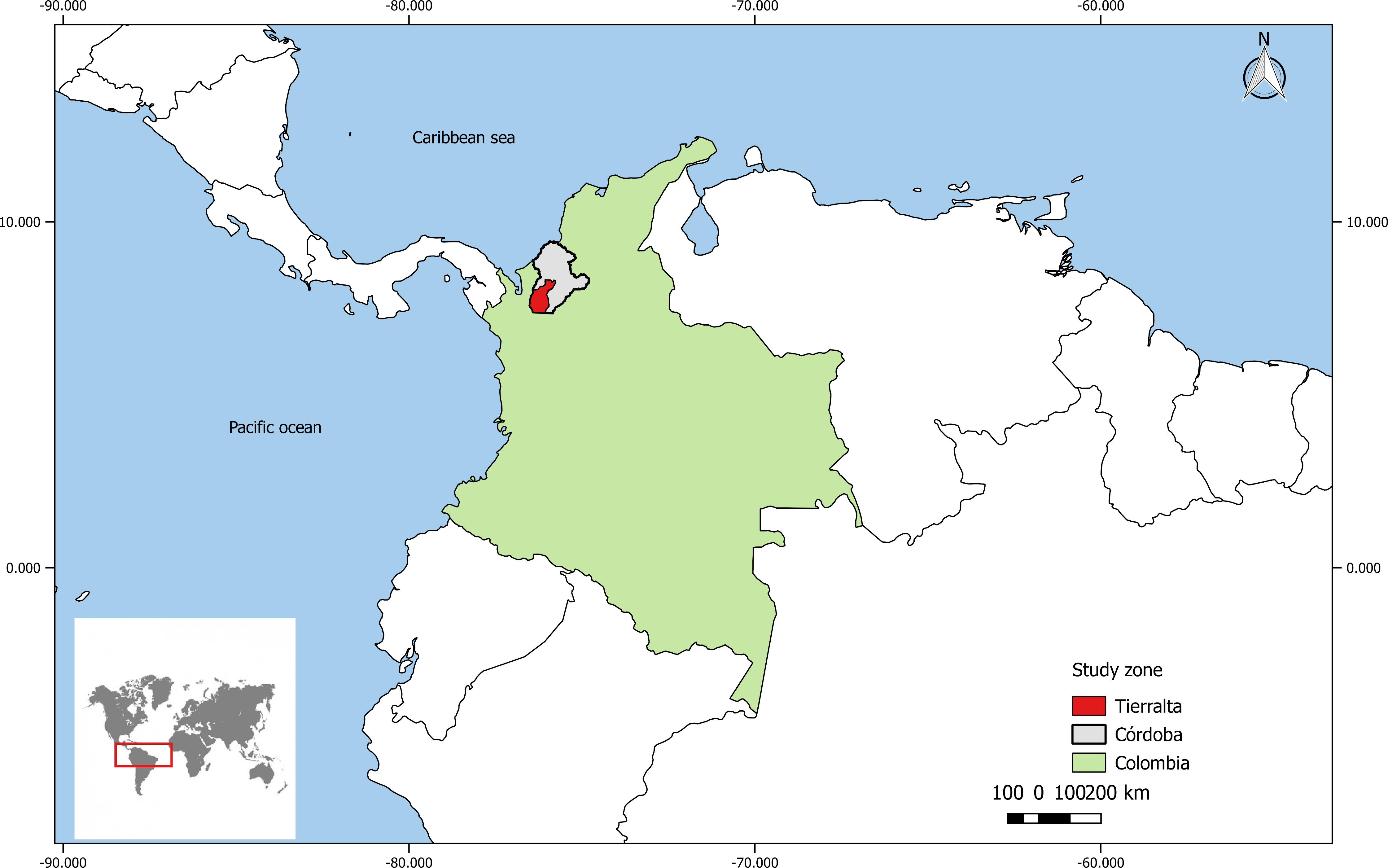

Supplement: S1 Fig — (TIF) [file pntd.0008466.s001.tif]

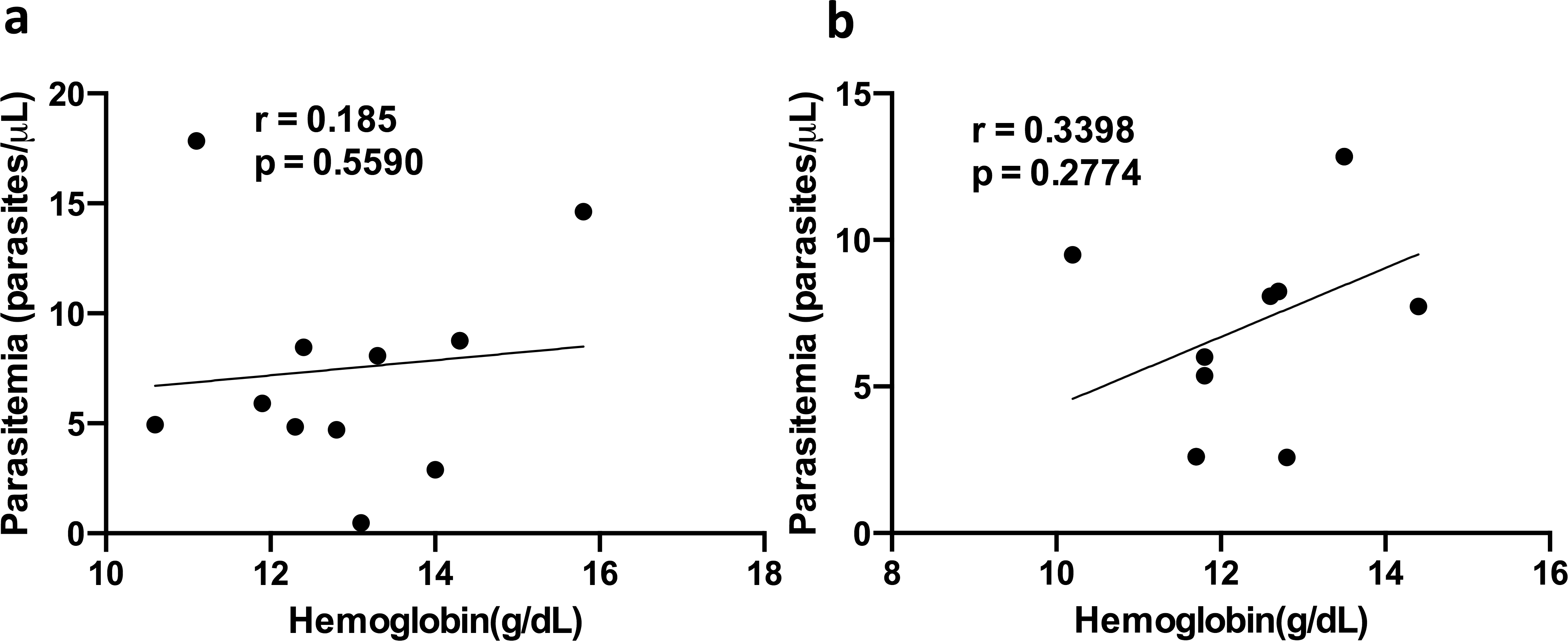

Supplement: S2 Fig — Correlation analysis of initial parasitemia (day 0) with hemoglobin of Colombian P. vivax (a) and P. falciparum (b) patients. Significance assessed by non-parametric Spearman correlation analysis. (TIF) [file pntd.0008466.s002.tif]

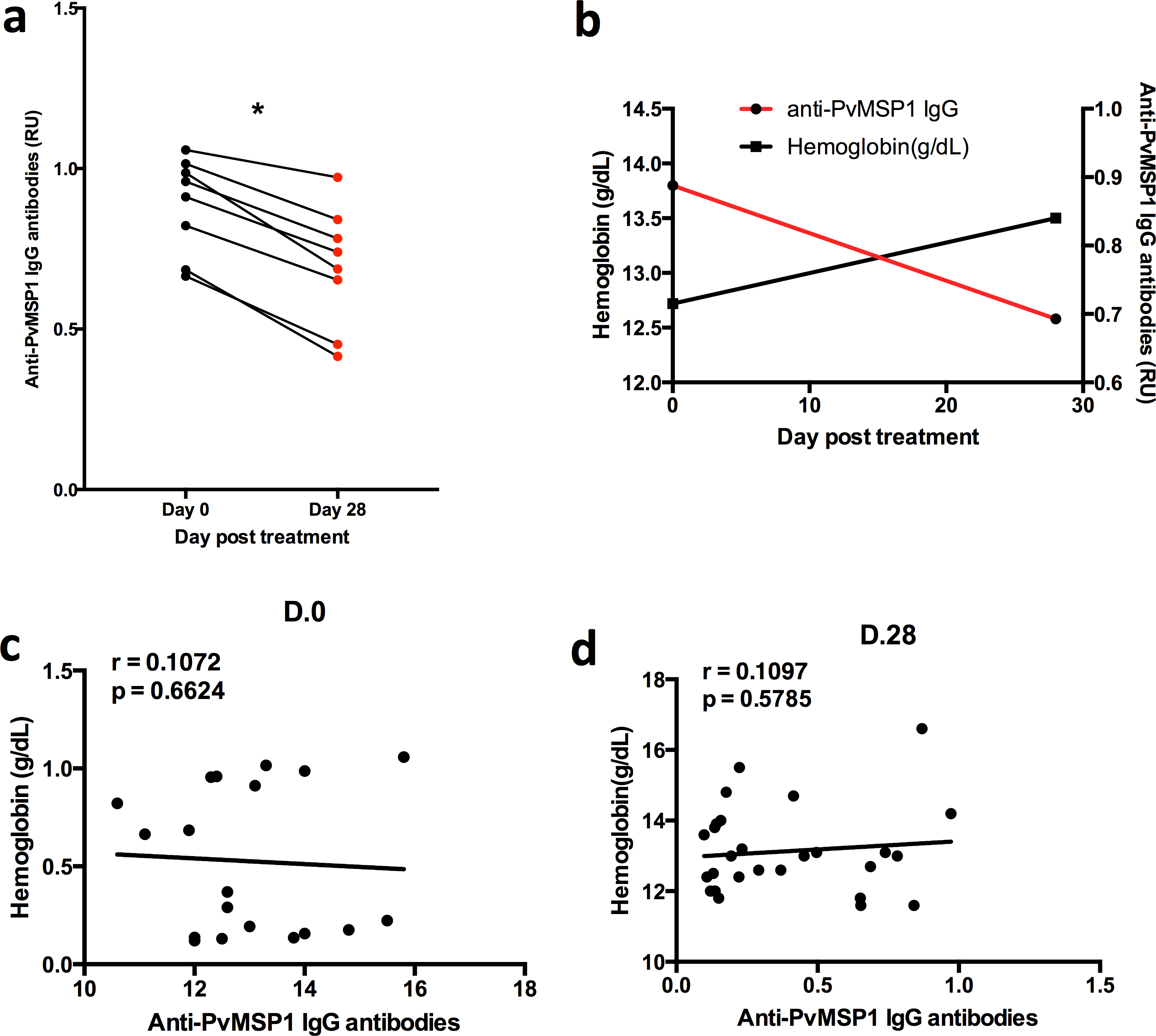

Supplement: S3 Fig — (a-b) Longitudinal analysis of the dynamics of anti-P. vivax MSP1 IgG antibodies between day 0 and 28 post-treatment (a) and with hemoglobin (b). (c-d) Correlation analysis of anti-P. vivax MSP1 IgG antibodies for day 0 (c) and 28 (d) post-treatment with hemoglobin. Significance assessed by Unpaired Student T-test (a) and by non-parametric Spearman correlation analysis (c-d). *p≤0.05. (TIF) [file pntd.0008466.s003.tif]

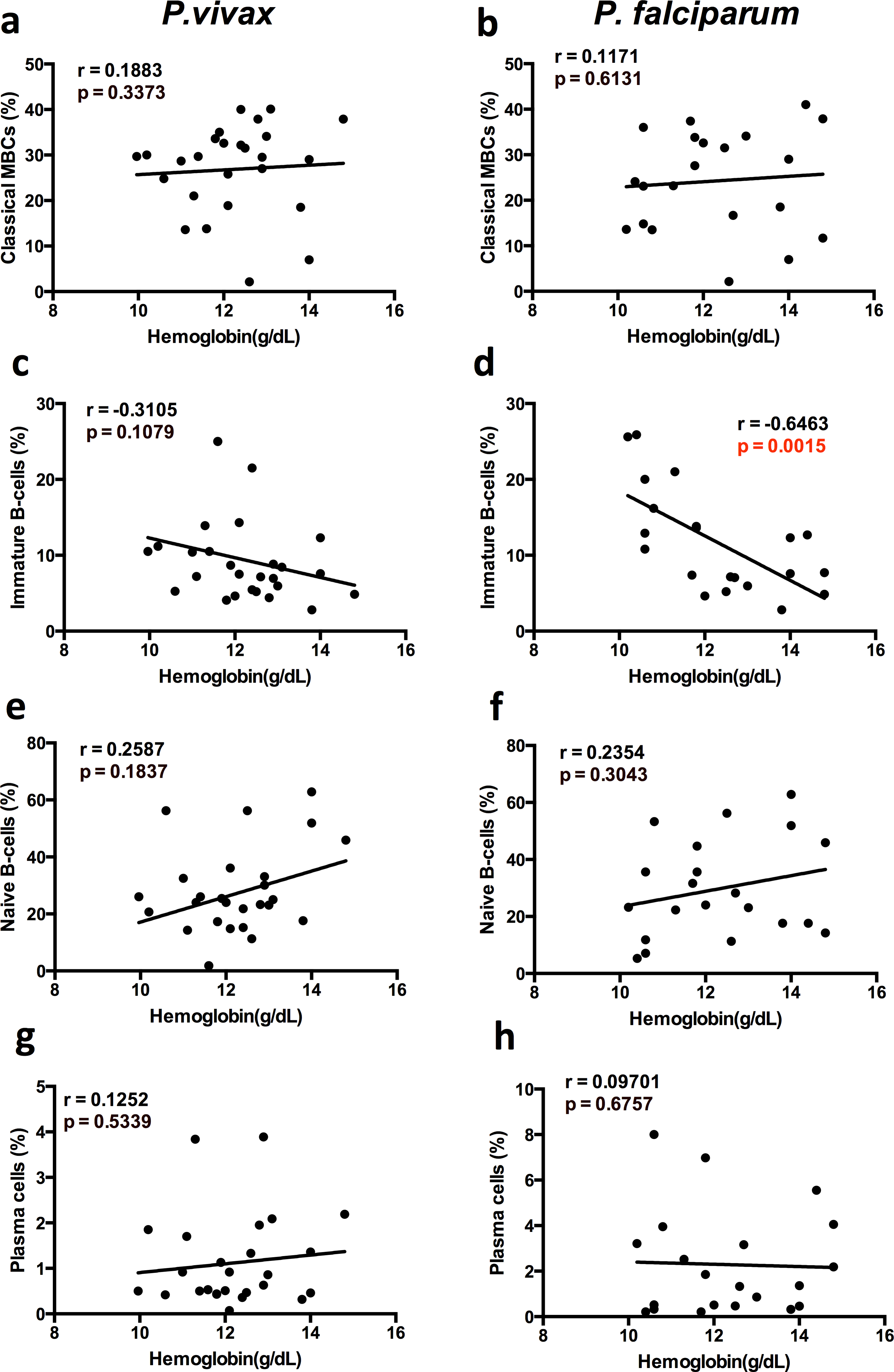

Supplement: S4 Fig — Correlation analysis of hemoglobin levels and classical memory B-cells (a-b), immature (c-d), naïve B-cells (e-f) and plasma cells (g-h) from PBMCs of P. vivax (a,c,e,g) and P. falciparum (b,d,f,h) patients at the two time points with lowest hemoglobin. Significance was assessed by non-parametric Spearman correlation analysis. (TIF) [file pntd.0008466.s004.tif]

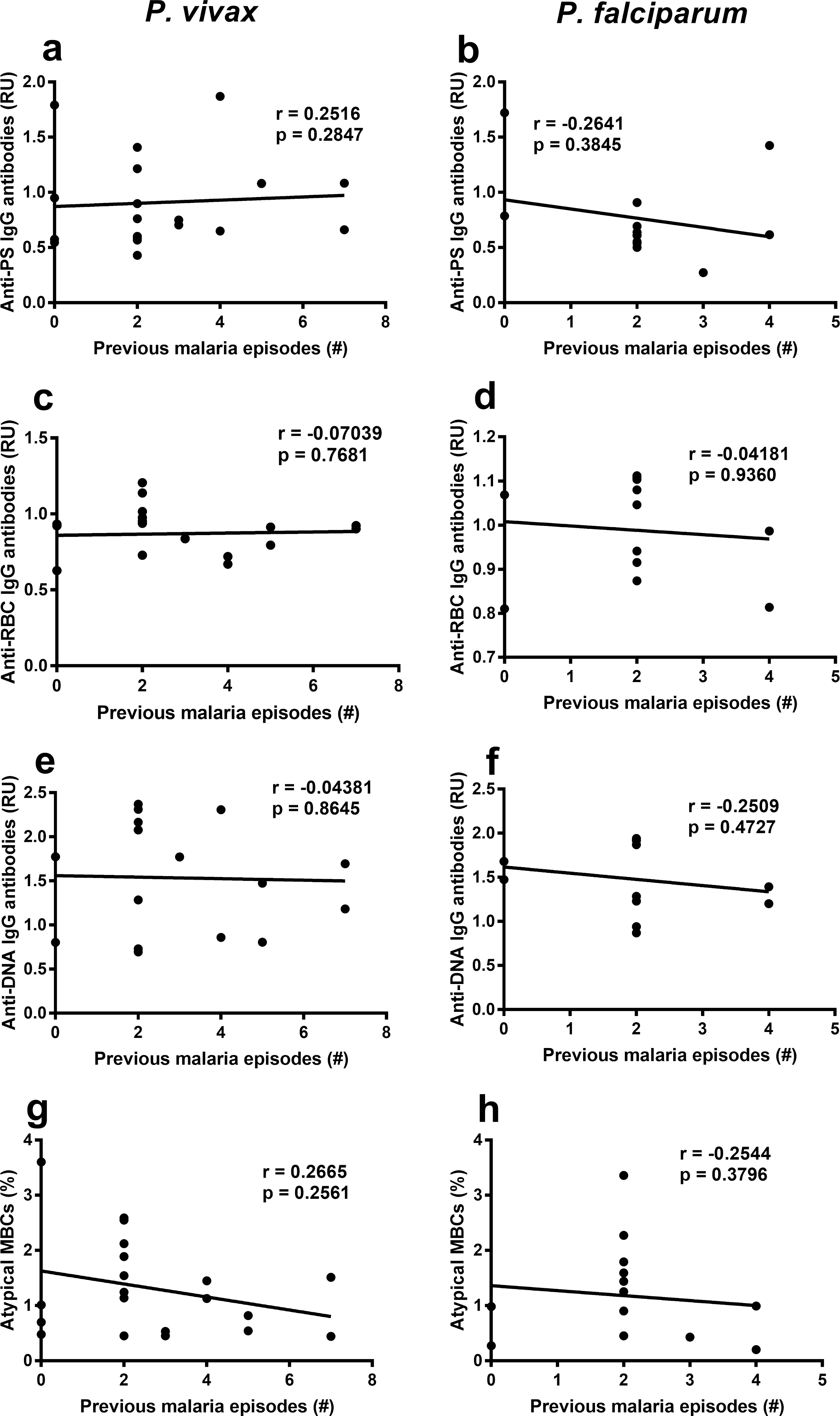

Supplement: S5 Fig — Correlation analysis of previous malaria episodes with anti-PS (a, b), anti-RBC lysate (c, d) or anti-DNA (e, f) IgG antibodies or atMBCs (g, h) at anemic time points between P. vivax (a,c,e,g) and P. falciparum (b,d,f,h) patients from cohort 1. Significance was assessed by non-parametric Spearman correlation analysis. (TIF) [file pntd.0008466.s005.tif]

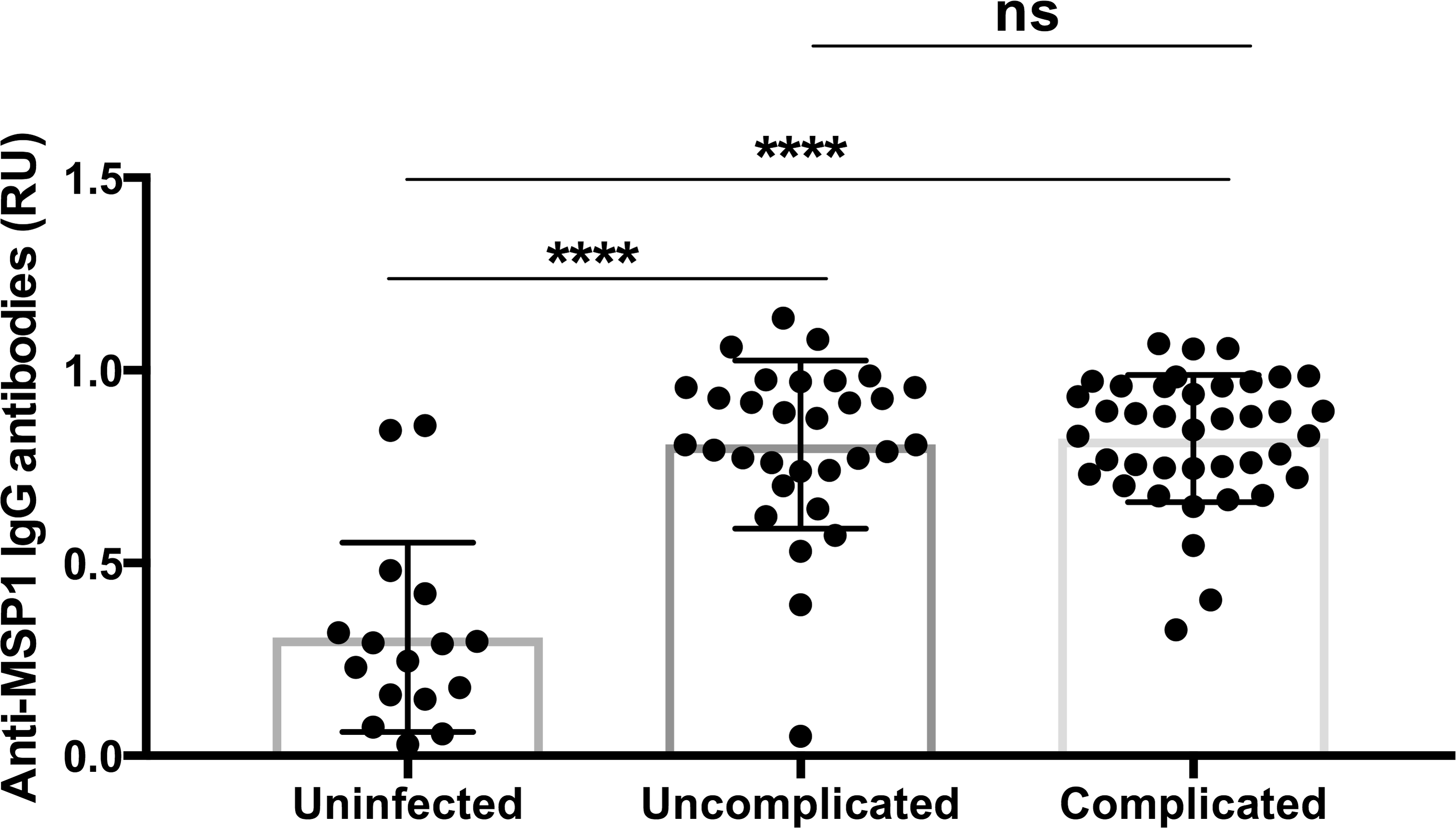

Supplement: S6 Fig — Bar graphs representing the levels of anti-P. vivax MSP1 antibody levels from plasma of uninfected controls and P. vivax patients with uncomplicated or complicated infection. Significance assessed by One-way Anova. (TIF) [file pntd.0008466.s006.tif]
