# Supplementary material for: Atypical memory B-cells and autoantibodies correlate with anemia during Plasmodium vivax complicated infections
Source: PLoS Negl Trop Dis. 2020 Jul 20;14(7):e0008466. doi: 10.1371/journal.pntd.0008466 (PMC7392348; doi:10.1371/journal.pntd.0008466)
Supplement: Attachment — Submitted filename: Reviewer_responses_PNTDs_060220_final.pdf [file pntd.0008466.s007.pdf]

<u>Responses to Reviewer's Questions</u>

We would like to thank the reviewers for their comments, and the Editors for the opportunity to revise and improve our manuscript. Please see our point-by-point responses to the reviewer comments below. Also, note that all text line indications are marked for their location in the "marked up" version of the manuscript text.

Reviewer #1: (No Response)

Reviewer #2: The manuscript by Rivera-Correa et al. points to an association of anti-PS autoantibodies and atypical memory B cells with anemia in two different cohorts of P. vivax patients, particularly in complicated P. vivax ones. They have now extended their previous work by showing a strong inverse correlation between different IgG autoantibodies (anti-PS, anti-DNA, and anti-RBC) and atypical memory B cells (atMBCs) with hemoglobin in both P. vivax and P. falciparum patients over time. Moreover, they reported a stronger association between hemoglobin levels, atMBCs, and anemia in complicated P. vivax patients compared to uncomplicated ones. Despite anemia is an important clinical manifestation of P. vivax infection, little is known regarding the mechanism underlying this disease outcome, thus the work is an important contribution to the field. However, there are several points that must be addressed to make it clearer and more robust.

- The two main species that cause human malaria, P. falciparum and P. vivax, present biological particularities influencing the pathogenesis of severe infections, mainly anemia. The destruction of uninfected RBCs plays a crucial role in the etiology of vivax malaria, more than in P. falciparum infections. Thus, we expected an increase of anti-PS autoantibodies during vivax malaria when compared to levels in P. falciparum-infected patients.
However, according to figure S2, the authors observed similar levels of autoantibodies (anti-Ps and anti-RBC) between P. vivax and P.falciparum Colombian patients. Do the authors have some explanation for such findings?

We thank the reviewer for the insightful comments and detailed review. In the new version of the manuscript, we have included an explanation for the surprising results of Figure S2 (now new Figure 1).

Our previous work in mice (Fernandez-Arias et al., Cell, Host & Microbe, 2016), established a mechanism for the process of elimination of uninfected RBC during malaria. The elimination of uninfected RBCs depends directly on two factors: the exposure of PS on the surface of the RBC and the binding of anti-PS antibodies to it. A similar mechanism probably occurs in malaria patients, where if the exposure of PS was higher in RBCs of P. vivax patients may explain that, with similar levels of anti-PS, the elimination of RBC is higher in P. vivax.
This point has been further discussed in the discussion starting in line 693

Interestingly, levels of antibodies against RBC were lower than those verified for PS (please see fig. S2).

In this type of study it is only possible to compare the reactivity to a particular antigen or group of antigens between different plasma samples. However, it is not possible to compare the reactivity of a particular plasma sample to different antigens. This limitation is based on the fact that the characteristics of each ELISA used for each particular antigen are different. The different efficacy in the coating of antigens to the plate and different background of each assay, probably accounts for most of this variability. It is not possible to compare a pure antigen, such as PS, to a mix of antigens in RBC that are 'diluted' among many non-antigenic components of the lysate.

In the same direction, levels of atMBCs were not different between patients infected with P. falciparum and P. vivax (Fig. S4). Those unexpected results in relation to the biological features of both parasite species
were poorly discussed and deserve to be properly addressed. Moreover, I strongly recommend the authors to include figures S2 e S4 as part of the Results section, adjusting other figures to make the results more concise and direct (please see my comments below).

Thank you for this observation. The similar levels of atMBCs in P. falciparum and P. vivax patients may be explained by similar levels of anti-PS, as observed, but different levels of PS exposure in RBC.

Figures S2 and S4 have been incorporated to new main Figures 1 and 4 respectively. These results have been more thoroughly discussed in the Discussion session line 693

- The pivotal novelty of the manuscript is the analysis of complicated malaria patients regarding their anemia status. However, it is not clear the criteria used to determine anemia and its classification according to the intensity as mild, moderate or severe.
How many patients presenting complicated manifestation of vivax malaria had anemia (mild, moderate or severe)? Such information is essential to understand the relationship between anemia and autoantibodies and atMBCs.

To address this concern, new Table 4 has been added detailing anemia status of all patients based on WHO criteria.

- The Introduction Section is too repetitive and, in my opinion, needs to be more focused, pointing the main concerns. I advise the authors to define the hypotheses to be tested in relation to the two cohorts evaluated in the study. This makes it very easy to understand the results and develop an appropriate discussion.

Introduction has been modified to clearly define our hypotheses.

- I strongly recommend authors to supply titles and statistical analyses for Tables. Currently, nothing is provided. Some are impossible to understand without such information.

Titles and statistics have been added to tables.

- The small sample size of patients enrolled in the Cohort 1 is worth addressing. This limitation of the research should be addressed in the Discussion aiming to explain the obtained results.

This has been addressed in the discussion. See line 655

Reviewer #3: The objectives of the study are well articulated however a hypothesis is not clearly stated. The study aims to test whether an association previously found in P. falciparum also exists in P. vivax, but using two different designs (longitudinal and cross-sectional) and additional types of patients. The population was clearly described and appropriate for the objetives.
The study was exploratory and the sample size was not large but it appeared sufficient to ensure adequate power to address some but not all objectives. A sample size or power calculation was not done for a primary analysis (which was not defined).
Some correlations or dot plots had few points and that is probably the reason while statistical significance was not achieved in some of the secondary analyses.

The laboratory methods require more clarification: the ELISA for autoantibodies does not indicate the use of negative controls and the serum dilution is quite low (1/100), therefore there is concern about specificity of the response vs background response.

We thank the reviewer for the insightful comments and detailed review. The ELISA method for autoantibodies has been validated before using multiple comparisons of healthy versus malaria samples (Rivera-Correa et al., Scientific Reports, 2019 and Rivera-Correa et al., eLife, 2019).

Equally in this study, we included a healthy USA control sample to assess background in every ELISA plate, in addition to endemic uninfected controls for each cohort.
The negative USA control had the following range for the different ELISAs:
RU anti PS (0.102- 0.164)
RU anti RBC (0.212- 0.243)
RU anti DNA (0.154-0.181)
RU anti MSP1 (0.106-0. 135)
This has been further clarified in the methods section, see line 275

The statistical analysis used are quite simple but incompletely described. In methods it says that correlation analysis was Spearman but later they report Pearson in one occasion; Anova is not mentioned but later used, etc - revise consistency.

Thank you for this observation. The statistical analysis used has been clarified across the manuscript for consistency.

The study received ethical approval from the Colombian IRB where patients were recruited.

Results</br></br>
-Does the analysis presented match the analysis plan?</br>
-Are the results clearly and completely presented?</br>
-Are the figures (Tables, Images) of sufficient quality for clarity?</br></br>

Reviewer #1: (No Response)

Reviewer #2:
The authors need to remove any discussion, description of methods, references or conclusion from the results.
Results section has been modified according to reviewer's suggestions.

They also need supply titles and, statistical analyses for Tables.

Titles and statistics have been added to tables.

Figures should be improved to highlight the main evidence. Please, consider joining some figure referring to related results.

Figures and figure titles have been modified and merged according to suggestions above. See new figure 1, 4 and 7.

Reviewer #3: The analysis matches to a large extent the objectives presented (there was no proper analysis plan, just some simple methods listed).
Results are in general clearly and completely presented but since a lot of correlations are done, it needs to be better explained how they are interpreted. It appears the weight is given to the p values (highlighting in red) but it is better to give weight to the correlation coefficient (and state what intervals are considered relevant), and the scatter plots, rather than to the P value in such Spearman analyses but this is not well explained. In some cases a significant p value might not be convincing when looking at the r or scatter plots, while in others not significant p values can still appear significant looking at the correlation plot, only that too few

samples were included. In general, however, the correlation coefficients are reasonable for the conclusions.

Thank you for this suggestion. We have modified the conclusions accordingly.

Some supplementary figures need to be polished in terms of format, e.g. spaces (between P. and falciparum or vivax), italics, etc. Tables ok.

Thank you for the observation. Figures have been adequately polished. Please note new Figure 1, 4 and 7 and new Supplementary figures 1-6.

Conclusions</br></br>
-Are the conclusions supported by the data presented?</br>
-Are the limitations of analysis clearly described?</br>
-Do the authors discuss how these data can be helpful to advance our understanding of the topic under study?</br>
-Is public health relevance addressed?</br></br>

Reviewer #1: (No Response)

Reviewer #2: Please, see the general comments above.

Comments mentioned above have been incorporated to the revised version.

Reviewer #3: In general, the conclusions are supported by the data presented.
The imitations of analysis are not clearly described and the discussion is rather short, this should be expanded. There is also no sufficienc discussion on the public health relevance of the findings or in how these data can be helpful to advance our understanding of the topic under study.

Conclusions have been modified to address these concerns.

**Editorial and Data Presentation Modifications?</br>
**
Use this section for editorial suggestions as well as relatively minor modifications of existing data that would enhance clarity. If the only modifications needed are minor and/or editorial, you may wish to recommend “Minor Revision” or “Accept”.

Reviewer #1: (No Response)

Reviewer #2: Minor comments:

- Title: Include "anti-phosphatidylserine antibodies" since only those specific antibodies have been studied.

Other autoantibodies in addition to anti-PS have been studied in their relation to anemia (Figure 7), including antibodies recognizing whole RBC lysate and anti-DNA which correlate inversely with hemoglobin in complicated P. vivax patients, while no correlation was found with anti-P. vivax MSP1 antibodies.

- Line 29: 'are' do not fit in this sentence.

Corrected in the new version of the manuscript. See new line 45

- Line 35: add Colombia before South America.

Corrected in the new version of the manuscript. See new line 51

- Line 70: this sentence needs to be supported by a reference.

Corrected in the new version of the manuscript. Reference has been added, please see line 89

- Line 72: the study conducted by Mourao et al. 2018 should also be cited here.

Corrected in the new version of the manuscript. See new line 91

- Line 104: The sentence "Very little is known about the pathogenesis of complicated P. vivax infections" must be removed.

Corrected in the new version of the manuscript.

-Line 109-111: Please, delete the affirmation since it was very premature and not directly related to the paper.

Corrected in the new version of the manuscript.

- Line 123: Figure 1 is necessary? In my opinion, it could be changed to Fig. S2 and S6, showing the levels of antibodies against PS, RBC, and DNA in each cohort. As I mentioned above, those are unexpected results that deserve attention and discussion.

Corrected in the new version of the manuscript. Old Figure S2 and S6 are new Figure 1. Old Figure 1 has been moved to supplementary as Fig. S1.

- Line 126: Plasmodium vivax is a species, not a strain. Please, correct it.

Corrected in the new version of the manuscript. See new line 179

- Line 133: In my opinion, it would be more informative if the authors mentioned here criteria used to determine anemia and its classification according to the intensity as mild, moderate or severe.

Corrected in the new version of the manuscript. New Table 4 indicates the classification of anemia intensity.

Besides this, it is also important to explain how the authors determined parasitemia.

Parasitemia determination was done by blood smear and confirmed by nested PCR. This is detailed in methods line 211

- Lines 127-132: Is there any overlap among patients from the two different cohorts?

Not in patients but yes in the Colombian controls. For PBMC analysis the Colombian controls from cohort 1 were used for cohort 2. See new line 289

-Table 1 is not adequate and must be improved to provide more information about cohorts and patients.

Tables have been modified.

How many patients were classified as presenting mild, moderate and severe anemia? According to the text, only one patient had levels of hemoglobulin above 7g/dL, what about the other two?

Anemia classification has been added in new table 4

- Line 137: "(hemoglobin ≤ 7gr/dL)" – remove "r"

Corrected in the new version of the manuscript. see line 217

- Line 141: Please, mention here what were the excluded infections.

The following infections were excluded: Dengue, brucellosis, leptospirosis, salmonellosis, rickettsial disease and mixed Plasmodium infection.
This has been added to the methods section, see line 222

- Lines 141-143: Wouldn't it be better to use individuals who have never been exposed to malaria?

Uninfected Colombian controls from cohort 1 were all from a non-endemic Colombian city (Monteria) where the population has rarely been exposed to malaria. These controls were also used for the PBMC analysis of cohort 2.

For cohort 2, endemic Colombian controls were also used in the antibody analysis. All were confirmed to be PCR negative for Plasmodium infection.

This information is now included in methods section, see lines 182 and 223.

Did the authors consider the last species infecting everyone?

Unfortunately, we do not know the last species that had infected everyone. This information is complicated to accurately obtain at the endemic site.

In an endemic area, how can you be sure that those individuals did not have any infection along the six months period?

All of the controls used for cohort 1 were from the non- malaria endemic Colombian city of Montería.

Endemic controls from cohort 2 were verbally asked about previous infections and confirmed no previous infection along the six-month period.

Did the authors also perform the diagnosis to discard a subpatent infection (as they could be asymptomatic individuals)?

Yes, all controls were considered negative as confirmed by nested PCR. See methods line 224

- Line 150: table 1 - Please, change "(11)" to (n = 11) and (n = 9)

Corrected in the new version of the manuscript.

- Line 156: table 2 – Did you evaluate the number of previous malaria episodes? In my opinion, this variable is important to understand different aspects of vivax malaria including the outcome of mild or severe disease.

Thank you for this suggestion, as we had not considered this variable.

We performed a non-parametric correlation analysis of previous malaria episodes with all autoantibodies (PS, RBC, DNA) or atypical memory B-cells and found no significant relationship between them for neither P.vivax nor P.falciparum. Please see new supplementary Figure S5.

In this table, the authors should also include other information that was evaluated and is also important to characterize patients such as GPT, GOT, bilirubin direct, bilirubin total, creatinine, glycemia, platelets, leukocytes, erythrocytes, hematocrit, Hg. Do the authors have data about reticulocytes count? If yes, please include it.

Unfortunately, these clinical parameters were not taken for Cohort 1.

- Line 174: Which was the temperature used for blockage? Please, describe the blocking buffer.

Corrected in the new version of the manuscript. Assays were blocked at 37 °C with PBS 1X 3% BSA buffer. See Line 268

- Line 176: Please, mention the solution in which the secondary antibody was diluted.

Corrected in the new version of the manuscript. The solution used was PBS 1X 0.5% BSA. See Line 270

- Line 178-180: Please, indicate at least one reference supporting this method to determine autoantibody levels.

The ELISA method for autoantibodies has been validated before using multiple comparisons of healthy versus malaria samples (Rivera-Correa et al., Scientific Reports, 2019 and Rivera-Correa et al., eLife, 2019).
Corrected in the new version of the manuscript. See new line 278

- Line 198: For figure 2, the authors did not perform any statistical analysis. As they compared the measurements of Hb and antibodies for the same patients, before and after nadir, the statistical test must be paired. Please review the analysis and complete this information.

Thank you for this suggestion. Statistical analysis for this figure is now included in the revised version of the manuscript.

- Line 201: replace "form" to "from"

Corrected in the new version of the manuscript.

- Line 207-210: Please, clarify the number of studied individuals: "P. vivax (n =11) or P. falciparum (n = 9)" means 20 patients, not 17 as pointed. "(n = 99 unique samples)" – is this the total number of samples collected in different moments? Please, confirm this.

Yes, 99 is the total number of samples collected in different moments.
Clarified in the new version of the manuscript. See line 311

- Line 216: Since the authors are talking about proportion, it is better to mention the percentage in parenthesis rather than numbers (8/11 and 5/9).

Corrected in the new version of the manuscript. See line 314

- Line 227: Change figure legend to Dynamics of autoantibodies and hemoglobulin levels in Colombian P. vivax and P. falciparum patients. Note that several patients are not anemic according to WHO criteria.

Corrected in the new version of the manuscript. See new line 356

- Lines 235-236: This statement seems to be not clearly depicted in the graphs. Please, adjust the information accordingly.

Corrected in the new version of the manuscript.

- Line 243: In my opinion, the relationship between two variables is generally considered strong when their r value is larger than 0.7. This is not the case for values such as: 0.41, 0.48, 0.47, 0.50, 0.37. Therefore, the word strong should be removed.

Corrected in the new version of the manuscript.

- Line 251: What does "the two time points with lowest hemoglobin" mean? Please, clarify.

Explained in the new version of the manuscript. See Line 292

- Line 257: Correct to subpopulations.

Corrected in the new version of the manuscript.

- Lines 271: Delete "in".

Corrected in the new version of the manuscript.

- Line 301: What does it mean "possible relation"? There is a relation or not regarding the results presented.

Corrected in the new version of the manuscript.

- Lines 312-313: Change figure legend to Correlation analysis of autoantibodies with epidemiological and clinical parameters in P. vivax patients with uncomplicated or complicated malaria.

Corrected in the new version of the manuscript. See Line 504

- Line 355-356: Why did the n change from this assay to the latter one?

The n changed since PBMC samples were only available for this number of patients. See new line 289

- Line 408-411: I missed some discussion about the relationship between reticulocytes and P. vivax. How can anti-PS autoantibodies influence on P. vivax infection, as this species exclusively invades reticulocytes?

Discussion has been added in the new version of the manuscript. See Line 643

- Lines 416-417: There is some evidence in the literature showing increased anti-PvMSP1-19 levels, negatively correlated with a decrease in hemoglobulin levels see Sepulveda et al., 2016). Other P. vivax antigens have also been correlating to anemia as PvMSP3 (see Mourao et al., 2012). Consider discussing the results accordingly.

Thank you for providing those references. They have been incorporated to the discussion in the new version of the manuscript. See Line 655

- Line 420: remove South America since the main goal is not to evaluate geographical parameters but, outcomes related to P. vivax species that can be extrapolated to other patients living in vivax endemic areas.

Corrected in the new version of the manuscript.

- Line 450: "…atMBCs constitute a novel component with diagnostic and therapeutic potential for this syndrome." This statement should be moderated.

Corrected in the new version of the manuscript. See Line 738

- Figure 2: Add the word P. vivax above its corresponding graphs (a, c, e) and P. falciparum above b, d, and e. Moreover, correct the Y-axis of graph c (anti-RBC instead of anti-PS).

Corrected in the new version of the manuscript. See new Figure 2

- Figure 3: The same observation described in figure 2.

Corrected in the new version of the manuscript. See new Figure 3

- Figures 4 and 5 should be jointed as well as Figures 8 and 9.

These figures have been merged as suggested. Please see New Figure 4 and 7

Reviewer #3: Editorial suggestions as well as relatively minor modifications of existing data that would enhance clarity:
Abstract requires revising of the language (use of punctuation, selection of words, grammar, etc)

Corrected in the new version of the manuscript

Atypical Memory B cells do not need capital letters at the early paragraphs.

Corrected in the new version of the manuscript

In introduction reviese the use of words to avoid repetition of some terms.

Corrected in the new version of the manuscript

Line 70: introduce a reference at the end of sentence.

Corrected in the new version of the manuscript. Reference has been added, please see line 89

There are key relevant papers missing on the presence of atypical memory B cells and P. vivax malaria that should be added, one in Latin America and the other in Papua New Guinea.

Additional references have been added related to this point. See lines 665

Review the use of abbreviations throughout to avoid duplicating definitions.

Corrected in the new version of the manuscript.

Summary and General Comments</br></br>
Use this section to provide overall comments, discuss strengths/weaknesses of the study, novelty, significance, general execution and scholarship. You may also include additional comments for the author, including concerns about dual publication, research ethics, or publication ethics. If requesting major revision, please articulate the new experiments that are needed.

Reviewer #1: Anemia is a common symptom seen in both P. falciparum and P. vivax infections. It has long been known that the level of anemia is out of proportion to the number of erythrocytes that are lysed from parasite infection. Besides suppressed erythropoiesis there is a large component of lysis of uninfected erythrocytes which contributes to this anemia. This group, and others, have made significant recent strides in determining the cellular mechanisms behind this phenomenon in both mouse models and P. falciparum. This manuscript extends those findings to P. vivax, where lysis of uninfected vs infected erythrocytes might be even more skewed towards uninfected.
This paper reports on two cohorts of P. vivax patients – one uncomplicated longitudinal cohort of both P. vivax and P. falciparum uncomplicated cases and one cross-sectional cohort comparing uncomplicated to complicated P. vivax infection. Although not overly innovative (the techniques are all identical to those used in previous studies in P. falciparum) the study is meaningful and impactful. The work had not previously been done in P. vivax and similarity to P. falciparum should not have been assumed.
The work is very nicely done. It is presented well and analysed appropriately. Several concerns are listed below:

Figure 2 – the number of time points on the x-axis is confusing as presented. The Methods state that samples were taken and admission and then at d7,14,21, and 28 days after. That should result in a total of 5 times points. Why are there 7 time points on these graphs?

The total number of time points for each patient is 5. The reason why 7 time points appear in the axis is because the data were analyzed considering the nadir as the reference time, which is reached within one or two weeks difference between patients.

Figure 2 shows that P. vivax has more anti-DNA autoantibodies than P. falciparum. This was also shown in the Barber et al JID paper and deserves comment in the Discussion section.

As shown in new figure 1 (old figure S2) we saw no differences in autoantibody levels between P. vivax and P. falciparum patients.

Barber et al JID reported higher levels of anti-PS antibodies in P. vivax compared to P. falciparum patients from Malaysia at enrollment. Accordingly in that study, patients with P. vivax had lower hemoglobin values than with P. falciparum infection hence possibly explaining this increase in anti-PS antibodies.

In our study, we focused our analysis for cohort 1 on anemic time points, controlling for hemoglobin, hence possibly explaining this difference in the results between the studies. Further comments have been added to the Discussion related to this point. See line 693

Figure 3 – the correlation of anti-DNA autoantibodies to anemia for P. falciparum is

quite different from the results seen in the paper previously published by this group in eLife. Comments should be made.

Thank you for this observation. It is true that our eLife study on first-time P. falciparum infections in non-endemic naïve individuals showed no correlation of anti-DNA antibodies with hemoglobin.

However in our other published study (Rivera-Correa et al Scientific reports 2019) of severe P. falciparum infections in Uganda children, we did see a correlation of anti-DNA antibodies with hemoglobin, just like this study. Altogether these results suggest that anti-DNA antibodies could promote anemia in infected individuals from endemic areas but not, or not as marked at least, during first-time infections.

This has been further discussed in Line 650

Correlations between autoantibodies and anemia are seen in uncomplicated P. vivax in Cohort 1, yet they are NOT seen in the UCM cases in Cohort 2. Is that because of the natural history of P. vivax disease and the correlation would have developed later in the disease process? Or due to correlations only being seen in Cohort 1 when looking at the two lowest Hb points? This should be discussed. A reader that does not spend significant time in the manuscript might be confused with the apparent contradiction. The difference was also not mentioned in Lines 435 and 436.

Thank you for this observation. This difference is most like due to using the anemic points during in UCM patients in cohort 1 compared to cohort 2. This has been further discussed in Line 710

The major contribution of this paper is extension of similar findings from P. falciparum to P. vivax. Given such, there should be at least a paragraph in the Discussion to compare and contrast the mechanisms in the two species. There might be hints of differences in anti-DNA autoantibodies. These should either be explained away or described in detail. There also are often numbers quoted that P. falciparum lyses 8 RBCs per every infected RBC, whereas P. vivax lyses 34:1. These numbers could either be explained as significantly different – or described as likely to be 'statistically insignificantly different'.

An additional paragraph has been added discussing these concerns Please see line 693

Minor:

Line 136 – hypoglycemia, not just 'glycemia'

Corrected in the new version of the manuscript.

Table 2 – Consistency is needed with "." vs "," in numbers as well as the number of significant digits reported.

Corrected in the new version of the manuscript. See new Table 2

Line 207 – does not add up

Corrected in the new version of the manuscript.

Line 296 66% vs 64% on line 135

Corrected in the new version of the manuscript.

Figure 2 – I believe that the right-hand y-axis is mislabeled in graph c – shouldn't this be anti-DNA?

Corrected in the new version of the manuscript. See new figure 2c.

Line 326 Is that meant to be "complicated" rather than "uncomplicated"?

This is the initial analysis of the uncomplicated group in cohort 2. For the analysis of complicated malaria see line 545

Figure 9 – x-axes should be harmonized. The graph for Atypical B cells goes from 6 to 14 while the others go from 5 to 20

Thank you for this observation. Corrected in the new version of the manuscript. Figure 8 and 9 have been combined into new figure 7 according to reviewer indications. See new figure 7.

Reviewer #2: Although the manuscript represents an important contribution to the field of knowledge, some flaws should be considered by the authors during resubmission:
(i) absence of a clear criterion for the characterization of anemia (mild, moderate and severe) in the study population difficult the interpretation of the role of anti-PS autoantibodies and atMBCs during malarial anemia

Please see new Table 4 for anemia severity in both cohorts.

(ii) absence of an adequate comparison and discussion of the immunopathological processes of anemia during P. falciparum or P. vivax malaria, also difficult the interpretation of the results.

Further discussion has been added regarding this topic. Please see lines 693

Reviewer #3: Novelty: the immunopathological mechanisms being investigated by the paper, which generate from seminal work by the senior author's lab (relationship betweein autoimmune antibodies particularly anti-PS, anemia, atypical B cells and malaria) are less innovative as similar studies have already been done in P. falciparum and P. vivax. The novelty now is related to the longitudinal nature of the analysis and the association with symptomatology and severity to P. vivax that is a more neglected human infection.

PLOS authors have the option to publish the peer review history of their article (what does this mean?). If published, this will include your full peer review and any attached files.

If you choose "no", your identity will remain anonymous but your review may still be made public.

Do you want your identity to be public for this peer review? For information about this choice, including consent withdrawal, please see our Privacy Policy.

Reviewer #1: No

Reviewer #2: Yes: Erika Martins Braga

Reviewer #3: No

Figure Files:
While revising your submission, please upload your figure files to the Preflight Analysis and Conversion Engine (PACE) digital diagnostic tool,https://pacev2.apexcovantage.com. PACE helps ensure that figures meet PLOS requirements. To use PACE, you must first register as a user. Then, login and navigate to the UPLOAD tab, where you will find detailed instructions on how to use the tool. If you encounter any issues or have any questions when using PACE, please email us at figures@plos.org.

Data Requirements:
Please note that, as a condition of publication, PLOS' data policy requires that you make

available all data used to draw the conclusions outlined in your manuscript. Data must be deposited in an appropriate repository, included within the body of the manuscript, or uploaded as supporting information. This includes all numerical values that were used to generate graphs, histograms etc.. For an example see here: http://www.plosbiology.org/article/info%3Adoi%2F10.1371%2Fjournal.pbio.1001908#s5.

Reproducibility:
To enhance the reproducibility of your results, PLOS recommends that you deposit laboratory protocols in protocols.io, where a protocol can be assigned its own identifier (DOI) such that it can be cited independently in the future. For instructions see http://journals.plos.org/plosntds/s/submission-guidelines#loc-materials-and-methods

---

In compliance with data protection regulations, you may request that we remove your personal registration details at any time. (Use the following URL:https://www.editorialmanager.com/pntd/login.asp?a=r). Please contact the publication office if you have any questions.
